# Meta-omics integration approach reveals the effect of soil native microbiome diversity in the performance of inoculant *Azospirillum brasilense*

**DOI:** 10.3389/fpls.2023.1172839

**Published:** 2023-06-29

**Authors:** Jessica Aparecida Ferrarezi, Heloísa Defant, Leandro Fonseca de Souza, João Lúcio Azevedo, Mariangela Hungria, Maria Carolina Quecine

**Affiliations:** ^1^ Laboratory of Genetics of Microorganisms “Prof. Joao Lucio de Azevedo”, Department of Genetics, Luiz de Queiroz College of Agriculture, University of São Paulo, Piracicaba, Brazil; ^2^ Embrapa Soybean, Soil Biotechnology Laboratory, Londrina, Brazil

**Keywords:** biofertilizer, recruitment, microcosms, microbiomics, PGPB, metadata, plant phenomics

## Abstract

Plant growth promoting bacteria (PGPB) have been used as integrative inputs to minimize the use of chemical fertilizers. However, a holistic comprehension about PGPB-plant-microbiome interactions is still incipient. Furthermore, the interaction among PGPB and the holobiont (host-microbiome association) represent a new frontier to plant breeding programs. We aimed to characterize maize bulk soil and rhizosphere microbiomes in irradiated soil (IS) and a native soil (NS) microbial community gradient (dilution-to-extinction) with *Azospirillum brasilense* Ab-V5, a PGPB commercial inoculant. Our hypothesis was that plant growth promotion efficiency is a result of PGPB niche occupation and persistence according to the holobiont conditions. The effects of Ab-V5 and NS microbial communities were evaluated in microcosms by a combined approach of microbiomics (species-specific qPCR, 16S rRNA metataxonomics and metagenomics) and plant phenomics (conventional and high-throughput methods). Our results revealed a weak maize growth promoting effect of Ab-V5 inoculation in undiluted NS, contrasting the positive effects of NS dilutions 10^−3^, 10^−6^, 10^−9^ and IS with Ab-V5. Alpha diversity in NS + Ab-V5 soil samples was higher than in all other treatments in a time course of 25 days after sowing (DAS). At 15 DAS, alpha diversity indexes were different between NS and IS, but similar in all NS dilutions in rhizospheric samples. These differences were not persistent at 25 DAS, demonstrating a stabilization process in the rhizobiomes. In NS 10^−3^ +Ab-V5 and NS 10^−6^ Ab-V5, Ab-V5 persisted in the maize rhizosphere until 15 DAS in higher abundances compared to NS. In NS + Ab-V5, abundance of six taxa were positively correlated with response to (a)biotic stresses in plant-soil interface. Genes involved in bacterial metabolism of riboses and amino acids, and cresol degradation were abundant on NS 10^−3^ + Ab-V5, indicating that these pathways can contribute to plant growth promotion and might be a result of Ab-V5 performance as a microbial recruiter of beneficial functions to the plant. Our results demonstrated the effects of holobiont on Ab-V5 performance. The meta-omics integration supported by plant phenomics opens new perspectives to better understanding of inoculants-holobiont interaction and for developing better strategies for optimization in the use of microbial products.

## 1 Introduction

The benefits provided by plant growth-promoting bacteria (PGPB) have been explored and applied for a wide range of cultivated plants (Gamalero and Glick, 2020; Wang et al., 2021; Ferrarezi et al., 2022; Gopalakrishnan et al., 2022; Guimarães et al., 2022). PGPB represent alternative and integrative inputs to minimize the use of mineral fertilizers and pesticides, reduce import dependency of farm products and, consequently, lower production cost to farmers (Canfora et al., 2021). In an integrated nutrient management system, PGPB play a key role in promoting direct and/or indirect effects in nutrient acquisition, abiotic and biotic stress tolerance, and control of pests and diseases (Fukami et al., 2018; Tang et al., 2020).

The genus *Azospirillum* comprises free-living soil bacteria that have been studied as important plant-growth promoters globally used in commercial inoculants (Hungria et al., 2010; Santos et al., 2021). The plant growth promotion attributed to *Azospirillum* includes a broad range of direct and indirect mechanisms, such as phytohormones production and modulation, nitrogen fixation, assortments of small-sized molecules, enhancement in membrane activity, proliferation of root systems, increased uptake of water, mobilization and solubilization of minerals, mitigation of environmental stressors and biological control of phytopathogens (Rodriguez et al., 2004; Bashan and de-Bashan, 2010; Fukami et al., 2018). It is known that the microbial native community may strongly be influenced by the role of inoculants. Moreover, combination of inoculants will not necessarily produce an additive or synergic effect, but rather a competitive process (Trabelsi and Mhamdi, 2013). Those interactions keep unclear and should be better investigated. For instance, Herschkovitz et al. (2005) have demonstrated that *A. brasilense* did not alter or disrupt the microbial system on maize field at the group-specific level. However, Renoud et al. (2022) demonstrated that *Azospirillum lipoferum* CRT1 inoculation significantly impacted the total bacterial community in the three evaluated fields, but only with the reduced formulation. Few studies have demonstrated the effect of native microbial community on *Azospirillum* spp. performance.

In the last decade, strains Ab-V5 and Ab-V6 of *A. brasilense* have been successfully used as commercial inoculants for cereals, pastures and rhizobia co-inoculation of legumes in Brazil (Santos et al., 2021; Guimarães et al., 2022). From a meta-analysis of the results obtained in 103 field trials with maize inoculated with these two strains in maize, Barbosa et al. (2022) confirmed significant increases in several growth parameters. However, despite good field results, an holistic understanding about the effects of inoculation with *Azospirillum* and other PGPB is still missing, and might help not only to explain inconsistent results (Ji et al., 2019), but also to optimize the benefits to plants. For Ab-V5, Vidotti et al. (2019) concluded that maize response to the strain was influenced by quantitative traits linked to root exudation, hormone balance and plant defense under N stress. The PGPB effect also influenced accuracy of performance prediction, making possible further genomic selection for characteristics involved in the Ab-V5-maize interaction (Vidotti et al., 2019).

Beneficial plant-microbe rhizospheric interactions have been explored to understand the drivers with potential to improve crop growth under nutrient deficient environments (Tang et al., 2020). Among the diverse ecological functions the soil microbiota provides, the nitrogen (N) and phosphorus (P) cycling are especially relevant on agricultural ecosystems. Metagenomics, with specific gene marks, allows a detailed comprehension of specific steps of N and P metabolism, offering a great opportunity to identify shifts related to management practices (Santos-Beneit, 2015).

The role of soil native microbial communities in plant fitness is undeniable, with microorganisms playing important roles in nutrient cycling, production of plant hormones, suppression of disease causing agents, pests and weeds (Mendes et al., 2011; Banerjee et al., 2018). The complex formed by the plant host genome and its associated microbiome is called holobiont (Vandenkoornhuyse et al., 2015). Under the evolution perspective, the holobiont has been evolving as a unit of adaptation and selection processes (Zilber-Rosenberg and Rosenberg, 2008, cited in Vandenkoornhuyse et al., 2015). The rhizosphere microbiome (rhizobiome) comprises a reservoir of ecological function that contributes to plant health and defense, responding in different ways to PGPB introduction (Visioli et al., 2018). From young maize plants, Visioli et al. (2018) observed that rhizosphere bacterial community influenced N- and sulfur (S)-cycling, production of plant growth hormones, and micronutrient bioavailability; additionally, antibiosis against phytopathogenic fungi could be enhanced by seed-applied inoculants. From holobionts, it is known that microbiome assembly is driven by multiple forces, including environmental selection and stochasticity. The first involves niche availability and microbial interactivity with species assortment and coadaptation (Oliphant et al., 2019). In the case of a microbial invasion represented by inoculation of a PGBB, it is unknown whether (and which) microbiome members are important for plant growth promotion in action with the inoculant. Considering both environmental selection and stochastic effects on the microbiome assembly, little is known about the dynamics of microbiome recruitment under plant-PGPB influence.

Microbial hubs were defined as taxa with high association with other members in a community, probably recruiting members named helpers (van der Heijden and Hartmann, 2016; Banerjee et al., 2018). As their removal results in important changes in structure and function of the communities, microbial hubs have also been proposed as keystone taxa (Banerjee et al., 2018; Monteoliva et al., 2022). Different studies including host genotypes, environments and developmental stages, indicate that plants share core microbiomes composed of taxa that may be globally important (Yeoh et al., 2017; Monteoliva et al., 2022). Knowing which taxa represent keystone and helper species may offer opportunities to optimize the soil microbiome to improve crop fitness and yield in a more effective and consistent way (Monteoliva et al., 2022). Recently, the term skopobiota has been coined to define “purposefully designed, exact and reproducible blend of multiple species of taxonomically identified microorganisms, whose components may act additively or synergistically to accomplish a predefined function in or on a specific environment” (Del Frari and Ferreira, 2021). The assembly of a plant growth-promoting consortium, hereafter mentioned as skopobiota, with reduced complexity of natural soil microbial communities has been shown useful to be studied and used (Monteoliva et al., 2022). In a similar approach, Peng et al. (2021) reported promising results in maize growth promoting by using a salt-tolerant bacterial skopobiota composed of three PGPB strains previously isolated from *Sonchus brachyotus* rhizosphere grown in saline soil. Their results indicate that inoculation of the skopobiota enriched maize rhizosphere microbiota compared with the non-inoculated control and it may promote beneficial bacterial groups in the rhizosphere of saline soil-exposed maize (Peng et al., 2021).

The dilution-to-extinction approach has been used in studies to assemble a minimal and effective skopobiota for lignocellulose degradation (Díaz-Garcia et al., 2021), degradation of keratinous materials (Kang et al., 2020), isolation of virus-hosts systems (Buchholz et al., 2021), ammonia-oxidizing bacteria (Aakra et al., 1999) and other fastidious microbes (Thrash et al., 2017). Garland and Lehman (1999) reported a non-linear relationship between functional richness and microbial inoculum density in six different models analyzed through the dilution-to-extinction method. Chen et al. (2020) also used a dilution-to-extinction approach to investigate the importance of soil bacterial diversity on plant productivity. To our knowledge, this study is the first one using dilution-to-extinction to understand plant-PGPB-microbiome interactions. Our main hypothesis is that the additive effect of recruited soil native microbial community members with an inoculant strain, in this case Ab-V5 of *A. brasilense*, is important for the persistence of the strain throughout time in bulk soil and plant’s rhizosphere. By reducing rare groups from native soil community, we hypothesized that bacteria taxa positively correlating with Ab-V5 in terms of relative abundance were ecologically important for the inoculant performance in the rhizosphere. Furthermore, when Ab-V5 was able to successfully occupy ecological niches and persist throughout the initial vegetative stage, we examined increases in relative abundance of soil-plant beneficial bacteria and metagenomic functions in response to PGPB persistence by integrating meta-omics data. The results from our study certainly open new venues to understand and maximize the use of PGPB towards a more sustainable agriculture.

## 2 Materials and methods

### 2.1 Plant growth-promoting bacteria and plant material

*Azospirillum brasilense* strain Ab-V5, used in commercial inoculants in Brazil, was supplied by the “Diazotrophic and Plant Growth-Promoting Bacteria Culture Collection of Embrapa Soja” (CollectionsWFCC #1213, WDCM #1054). It was routinely grown in DYGS medium (composition in g.L^-1^: glucose, 2; peptone, 1.5; yeast extract, 2; KH_2_PO_4_, 0.5; MgSO_4_.7H_2_O, 0.5; glutamic acid, 1.5; malic acid, 2; distilled water up to 1000 mL; pH 6.8 (Rodrigues Neto et al., 1986)) at 28°C, 150 rpm for 18 h. For experimental set-up, initial bacteria cell density was adjusted to 1 × 10^8^ CFU.mL^−1^ in 50 mL of NaCl 0.9%.

Conventional maize (*Zea mays* L.) hybrid seeds 30A37PW (Morgan Sementes e Biotecnologia) were used in the greenhouse trial.

### 2.2 Greenhouse experiment set-up

Sieved soil (mesh widths 4 mm) was used to construct a dilution-to-extinction microbial gradient. First, the soil was irradiated (IS - irradiated soil) with 50 kGy Cobalt-60 at Nuclear and Energy Research Institute (IPEN, Sao Paulo-SP, Brazil), according to the methodology described by Gore and Snape (2014) and McNamara et al. (2003). The microbial gradient was prepared by collecting a bulk sample of approximately 25 kg of cultivated soil (NS-soil native community, 5-10 cm of depth, inter-rows) at a maize field at the Department of Genetics (ESALQ-USP; 22°42’31.3”S 47°38’05.8”W, 535 m altitude, Köppen-Geiger climate type Cfa). A subsample of 250 g of NS was used to make a serial dilution (1:10 until 10^−9^) in autoclaved PBS (8 g NaCl, 0,2 g KCl, 1,44 g Na_2_HPO_4_ and 0,24 g KH_2_PO_4_ in 1000 mL distilled water, final pH 7,4) under shaking condition (180 rpm). Microcosms with 5 kg of IS were amended with 500 mL of dilutions 10^−3^, 10^−6^ and 10^-9^ of native soil (NS), and microcosms with 5 kg of NS and IS were used as controls (**Table 1**). Soil samples (IS and NS) were sent to fertility analysis at the Department of Soil Sciences (ESALQ-USP, Piracicaba-SP, Brazil) (**Supplementary Table 1**).

**Table 1:** Dilution-to-extinction gradient of the soil native microbiome with inoculation of *Azospirillum brasilense* strain Ab-V5.

Treatments*	Description
NS + Ab-V5	Native soil (NS) microbial community used to construct a microbial community diversity gradient amended with Ab-V5
NS 10^-3^ + Ab-V5	Dilution 10^-3^ of NS amended with Ab-V5
NS 10^-6^ + Ab-V5	Dilution 10^-6^ of NS amended with Ab-V5
NS 10^-9^ +Ab-V5	Dilution 10^-9^ of NS amended with Ab-V5
IS +Ab-V5	Irradiated soil with gamma-irradiation amended with Ab-V5

*NS – soil native microbiome, IS – irradiated soil by gamma-irradiation.

Maize seeds were surface disinfected by washing them in ethanol 70% (1 min), sodium hypochlorite 3% (1 min), ethanol 70% (1 min), and rinsed twice in sterile distilled water (Araújo et al., 2001). The seeds were then drained and left to dry at room temperature. After drying, they were submerged into the bacterial inoculum and incubated in shaking at 150 rpm for 30 min at room temperature. After 15 days of microbial communities stabilization in the microcosms, five inoculated maize seeds were manually sowed in microcosms containing NS and IS with and without NS dilutions. The randomized complete blocks experiment was conducted with four blocks (replicates) and five seeds per pot in a greenhouse at Department of Genetics (ESALQ-USP, Piracicaba-SP, Brazil) between February 27th and March 23rd, 2020. The microcosms were manually irrigated with 400 mL of distilled water every two days.

### 2.3 Sample collection

Samples of bulk soil were collected immediately after the NS microbial community gradient was amended to the soil irradiated by gamma-irradiation (NCI – native community inoculation), on the day of seed sowing (15 days after NCI, named 0 days after sowing - DAS), 15 and 25 DAS. Rhizospheric soil samples were collected 15 and 25 DAS, when most maize plants had 3-4 and 6-7 completely opened leaves (V3-V4 and V6-V7, respectively). After collection, the rhizospheric samples were stored in Falcon tubes containing 30 mL of autoclaved PBS and transported to the laboratory in an ice cooler box with ice packs. Rhizospheric samples were then submitted to ultrasonication (120 W, in iced water at 4° C, pulses of 30 seconds, with intervals of 30 seconds, 10 cycles; Simmons et al., 2018) to separate rhizosphere from root. All samples were stored at -20°C until DNA extraction.

### 2.4 Plant phenotypic traits assessment

Shoot and root samples of three plants per pot were collected (12 plants per treatment) 25 days after sowing (DAS), at the V6-V7 maize growth stage. Roots were carefully removed from microcosms containing soil, washed with water and stored in plastic microcosms with 20% ethanol solution for preservation. Root images were acquired by an Epson LA2400 scanner and processed using the WinRHIZO (Reagent Instruments Inc., Quebec, Canada), evaluating root length (RL, cm), root volume (RV, cm³) and root surface area (RSA, cm²). Shoot and root dry matter (SDM, g, and RDM, mg, respectively) were measured after drying samples at 50°C until constant weight (approximately 72h). Shoot-by-root ratio (SRR), specific root length (SRL, mg.cm^−1^) and surface area (SRSA, mg.cm^−2^) were calculated by dividing SDM (in mg), RL and RSA by RDM, respectively. Plant phenotypic data were analyzed following sparse Partial Least Squares Discriminant Analysis (sPLS-DA) (mixOmics version 6.10.9) using R software (version 1.1.456) (R Core Team). A *post-hoc* test (Fisher’s Least Significant Difference – LSD) was performed to determine pairwise differences among the means of each phenotypic attribute at 5% of significance.

### 2.5 DNA extraction

DNA extraction from bulk and rhizospheric soil samples was performed using DNeasy® PowerSoil Kit (Qiagen), following the manufacturer’s instructions. The integrity of the extracted DNA was evaluated in 1.0% (w/v) agarose gel electrophoresis with 0.5 X SYBR® Safe (Life Technologies) observed under UV light. DNA concentration was estimated by the spectrophotometer BioDrop µLITE (BioDrop, Cambridge, UK).

### 2.6 Quantification of PGPB abundance by qPCR

DNA was extracted from an inoculum of Ab-V5 (genome size: 6.934 Mbp; Hungria et al., 2018) in DYGS medium by DNeasy Blood and Tissue kit (Qiagen, Valencia, CA, USA), according to the manufacturer’s instructions. Fragments of the 16S rRNA gene (1.4 kbp) were generated by PCR from environmental genomic bacterial DNA using the methodology described by Heuer et al., 1997. These templates were quantified by spectrophotometer BioDrop µLITE and used for genome and gene copy number equivalent estimation, respectively (Staroscik, 2004). Standard curves and the limit of quantification (LoQ) for each template was determined by qPCR based on serial dilution from 10^7^ to 10² copy number. The primers sets used were: AzoR2.1R (GCATGCCCAGTACTGCAAGTC) e AzoR2.1F (CGCCACCATGCGATCAA), which detects a specific region of approximately 200 bp in a non-coding region specific of *A. brasilense* (Stets et al., 2015); 534F (CCAGCAGCCGCGGTAAT) and 783R (ACCMGGGTATCTAATCCKG), which are universal 16S rRNA primers (Rastogi et al., 2010).

The reactions were performed at a final volume of 12.5 µL in 96-well plates (SSIBio, UltraFlux® PCR plates, catalog ID 3450-00), containing 6.25 µL of GoTaq qPCR Master mix 2X (Promega; catalog ID A6001), 0.25 µL of each amplification primer (reverse and forward, 10 nM), 0.125 µL of CXR reference dye (Promega), 2 µL of DNA (20 ng of total DNA) templates and Milli-Q DNase-free water. Milli-Q DNase-free water was used as a negative control. The thermocycler (Applied Biosystems 7300 Quantitative PCR System) was programmed as follows: first step at 95°C for 5 min, a step of 35 cycles composed of 30 s at 95°C and 45 s at 60°C, and a final step of dissociation curve (single cycle of 15 s at 95°, 30 s at 60°, and 15 s at 95°). Cycle threshold (Ct) values obtained for each sample of known genome equivalent or gene copy number were used to determine linear regression models for Ab-V5 and 16S rRNA gene copies and the Ct values obtained were interpolated with the standard curves to estimate Ab-V5 genome equivalent and 16S rRNA gene copy number per g of soil. The log_10_ number of 16S rRNA gene copies was analyzed by LSD test at 5% significance level using R software.

### 2.7 16S rRNA metataxonomic sequencing

The composition of the microbial community was determined with high throughput sequencing (MiSeq Illumina platform, 2x250 bp) of the V3-V4 region of the 16S rRNA gene at NGS Genomics Solutions (Piracicaba-SP, Brazil). The V3-V4 region was amplified with the primers 341F (CCTACGGGNGGCWGCAG) and 785R (GACTACHVGGGTATCTAATCC) (Klindworth et al., 2013). DNA concentrations in the samples were adjusted to 10 ng/µL using a BioDrop spectrophotometer and the PCR reactions with 2.5 μL of 10× buffer, 1 μL of 50 mM MgCl, 1 μL of 10 mM dNTPs, 0.5 μL of 10 μM forward and reverse primers, 0.5 μL of 5 U.μL^-1^ Taq Platinum – PCR (Thermo Fisher Scientific, Waltham, MA, USA) and DNA-free water - 14 μL, in a total volume of 25 μL per reaction. Gene library was prepared with the cycles of 95°C for 3 min, followed by 25 cycles at 95°C for 30 s, 50°C for 30 s, 72°C for 30 s, and a final extension step at 72°C for 5 min. Subsequent DNA purification of the amplicon was performed using AMPure XP beads (Beckman Coulter, Brea, CA, USA) and verified on agarose gel. Similarly, the adapters were added by synthesis, followed by another purification with AMPure XP beads and confirmed on an agarose gel electrophoresis. The amplicon pool was normalized using quantification by qPCR with the KAPA Illumina quantification kit (Roche, Basel, Switzerland). The amplicon average size was 444 bp. Full description of samples for 16S rRNA metataxonomics is available in **Supplementary Table 2**. The data generated in this study is available at Zenodo (doi: 10.5281/zenodo.6359961) (**Supplementary Table 3**).

### 2.8 Metataxonomic sequencing data analysis

Demultiplexed paired-end sequencing FASTQ files were processed with tools from USEARCH pipeline v.11 (Edgar, 2010) and Ribosomal Database Project (RDP) (Cole et al., 2014). The raw reads were pre-processed for quality control, with adapters and low-quality bases trimmed, and data filtered. The main criterium for trimming was low-quality bases at 3′ extremes (quality score < 30). For filtering, the threshold of maximum error rate of 2% was established. After that, the reads were oriented to the RDP database to improve data annotation, submitted to dereplication (removal of redundant reads) and singletons removal (single reads or low-quality ones), and finally clustered at 100% identity to form zOTUs (zero-radius OTU). The zOTUs were counted per sample and annotated with RDP taxonomy information. At the end of the analysis, the files generated, namely zOTUs count table, metadata sheet and zOTUs mapping file (taxonomy), were submitted to analysis at MicrobiomeAnalyst platform (www.microbiomeanalyst.ca).

### 2.9 Whole metagenome shotgun sequencing and sequence annotation

Whole metagenomic sequencing (WMS) was used to sequence 12 DNA samples, selected after amplicon sequencing analysis for representing the treatments more responsive to *A. brasilense* Ab-V5 inoculation on growth promotion (**Supplementary Table 2**). All the samples refer to rhizospheric soils sampled at 15 DAS. The libraries were set using 100 ng of DNA of each sample (rhizosphere of treatments A, B and E collected 15 DAS) and TruSeq kit for library preparation (Illumina, SanDiego, CA, USA), according to the manufacturer’s protocol for WMS in an Illumina NextSeq 2000 platform (2×100 bp paired-end), at NGS Genomics Solutions (Piracicaba-SP, Brazil), with an average coverage of 20 mi clusters per sample. The WMS sequences were pre-processed and annotated with the web application server Metagenomic Rapid Annotations using Subsystems Technology (MG-RAST), pipeline version 4 (Meyer et al., 2008). The raw forward sequences were processed by quality control (QC) using SolexaQA software removing low-quality segments with “Dynamic Trim” method (Cox et al., 2010), according to the lowest Phred score of 15 and a maximum of 5 bases below the Phred score. After that, artificial replicate sequences and singletons were removed.

The 16S rRNA sequences were filtered from metagenomic data on MG-RAST, using the platform default tool. It used VSEARCH (Rognes et al., 2016) to check metagenome data against a reduced RNA database, 97% identity clustered version of the SILVA, Greengenes, and RDP databases to select the 16S rRNA sequences (MG-RAST, 2019). Then, the sequences were identified using a BLAST similarity search against the SILVA database, with e-value < 0.00001, identity 60%, a minimum abundance of 10, and representative hits.

The protein-coding region (features) identification was based on the protein database M5nr for a non-redundant integration of many protein databases (Wilke et al., 2012). The functional profiles were analyzed according to the SEED subsystem (Overbeek et al., 2014). Annotation parameters were those recommended by Randle-Boggis et al. (2016) using MG-RAST: maximum e-value cut-off of 1e^-5^ and minimum alignment length of 15 bp, while the minimal identity cutoff was 80%. Abundance profiles were determined using the ‘Representative Hit’ method and during the analysis the features annotated as eukaryotic and viral were removed. The metagenome dataset resulting from our QC and feature annotation are publicly available at MG-RAST database, project numbers mgl8686 (final digits: 05, 17, 35, 26, 23, 32, 14, 08, 29, 20, 11 and 38).

Nitrogen and phosphorus metabolism functions were identified according to subsystems (level 3 and Functions) annotation (https://pubseed.theseed.org/SubsysEditor.cgi). To identify significant changes in the metagenome profile, the tool Aldex2 was applied (Fernandes et al., 2014). The centered log_2_ ratios were set using Monte-Carlo samples of 128 and only those functions with p < 0.05 (FDR-adjusted) were plotted in the figures.

### 2.10 16S rRNA metataxonomics and metagenomics data integration

The Data Integration Analysis for Biomarker discovery using a Latent cOmponents (DIABLO) framework (Singh et al., 2017) in mixOmics (Rohart et al., 2017) was used to identify multi-omics signature discriminating the rhizospheric microbial communities under treatments NS + Ab-V5, NS 10^−3^ + Ab-V5 and IS + Ab-V5. First, we performed sparse Partial Least Squares Discriminant Analysis (sPLS-DA; function *block.splsda*), visualized the distribution patterns of individual samples in bidimensional space by block (i.e., omics approach; function *plotIndiv*), the correlation structure at the component level of each data set (function *plotDiablo*) and the correlations between variables represented on each treatment (function *circosPlot*). To obtain biologically relevant correlations, the threshold was set to 0.95 and only associations between metataxonomics and metagenomics variables exceeding this threshold were plotted. The correlation between variables is displayed as edges in blue (negative correlations) or red (positive correlations). The relative abundances of each selected variable are plotted as external lines in blue (NS + Ab-V5), ocher (NS 10^−3^ + Ab-V5) or gray (IS + Ab-V5).

## 3 Results

### 3.1 Plant growth-promotion in different treatments

**Figure 1:**
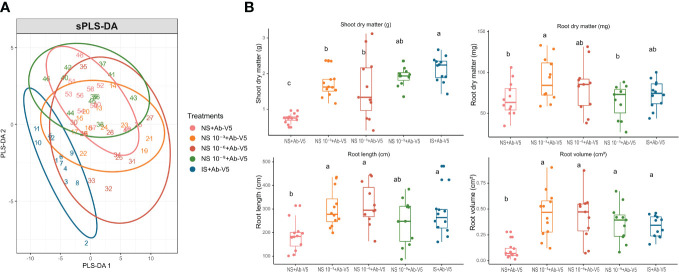
Effect of soil native microbial community gradient amended with *Azospirillum brasilense* Ab-V5 on maize phenotypic traits. **(A)** sparse Partial Least Squares Discriminant Analysis (sPLS-DA) among five treatments based on plant phenotypic traits evaluated 25 days after sowing bacterialized seeds (n=12). **(B)** Maize phenotypic traits evaluated 25 days after sowing bacterialized seeds (n=12). Different letters indicate statistically significant mean differences among treatments according to the LSD test (p-value < 0.05). NS, Natural soil (microbial community); NS 10^−3^, dilution 10^−3^ of NS; NS 10^−6^, dilution 10^−6^ of NS; NS 10^−9^, dilution 10^−9^ of NS; IS, Irradiated soil; Ab-V5, *Azospirillum brasilense* Ab-V5.

Based on the PGP traits, the Discriminant Analysis sPLS-DA was able to discriminate the treatments NS + Ab-V5 and IS + Ab-V5 in the first two dimensions, while NS 10^−3^ + Ab-V5, NS 10^−6^ + Ab-V5 and NS 10^−9^ + Ab-V5 showed more similarity among them (**Figure 1A**). Mean of shoot dry matter (SDM) in NS + Ab-V5 was significantly lower compared to all other treatments, whereas dilutions 10^−3^, 10^−6^ and 10^−9^ did not differ statistically and the treatment IS + Ab-V5 had highest SDM similar to NS 10^−9^ + Ab-V5. The treatment NS + Ab-V5 also had lower root length (RL) and volume (RV) compared to other treatments, while the means of other treatments did not significantly differ in these parameters. Means of root dry matter (RDM) of NS dilutions 10^−3^ and 10^−6^ + Ab-V5 were statistically similar to IS + Ab-V5; however, NS + Ab-V5 and NS dilution 10^−9^ + Ab-V5 had lower RDM compared to dilution 10^−6^ of NS + Ab-V5 (**Figure 1B**).

### 3.2 Quantification of 16S rRNA gene and Ab-V5 abundances by qPCR

**Figure 2:**
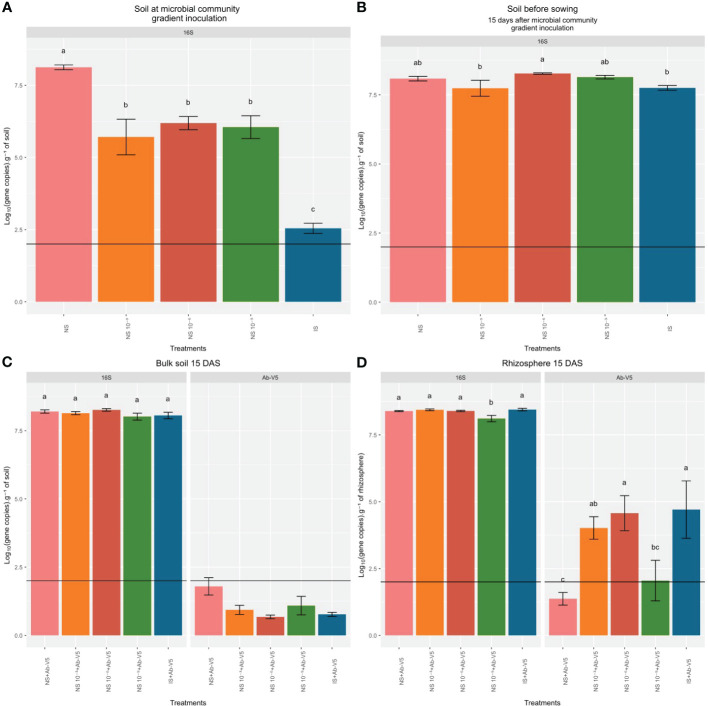
Quantification of 16S rRNA and *Azospirillum brasilense* Ab-V5 abundances by qPCR (means ± standard errors, n=8). **(A)** Bulk soil samples collected at inoculation of soil native microbial community gradient. **(B)** Bulk soil samples collected 15 days after inoculation of soil native microbial community gradient and before maize seeds with Ab-V5 were sowed. **(C)** Bulk soil samples collected 15 days after sowing (DAS) bacterized maize seeds in soil native microbial community gradient. **(D)** Rhizosphere samples collected 15 DAS bacterized maize seeds in soil native microbial community gradient. Horizontal black lines indicate the limit of quantification (LoQ) of Ab-V5 and 16S rRNA. Different letters indicate statistically significant mean differences among treatments according to the LSD test (p-value < 0.05). NS, Natural soil (microbial community); NS, dilution 10^−3^ of NS; NS 10^−6^, dilution 10^−6^ of NS; NS 10^−9^, dilution 10^−9^ of NS; IS, Irradiated soil; Ab-V5, *Azospirillum brasilense* Ab-V5.

The abundance of 16S rRNA per gram of soil in NS + Ab-V5 was approximately 10^8^, significantly higher than in the three dilutions series 10^−3^, 10^−6^ and 10^−9^ of NS, which were statistically equivalent (10^6^-10^5^ copies.g^−1^ of soil) and in turn higher than the IS + Ab-V5, of 10³, at microbial community gradient inoculation (**Figure 2A**). There was a stabilization of 16S rRNA abundance in 10^7^-10^8^ copies.g of soil ^−1^ between the moments of inoculating the microbial community gradient until 15 days later, *i.e.* at sowing of inoculated seeds (**Figure 2B**). The microbial abundance in bulk soil 15 DAS was totally stabilized around 10^8^ copies.g^−1^ of soil (**Figure 2C**). The Ab-V5 abundance in bulk soil at 15 DAS was below the LoQ (limit of quantification). In general, rhizospheric samples had statistically equal abundance of 16S rRNA (10^8^ copies.g^−1^ of soil), except for treatment NS dilution 10^−9^ + Ab-V5 with a lower mean 16S rRNA gene abundance (p-value < 0.05, LSD test). In IS and dilutions 10^-3^ and 10^-6^ of NS, the Ab-V5 abundance was statistically similar, approximately 10^4^ of NS and dilution 10^-9^ of NS, the mean Ab-V5 abundance in the rhizosphere was below the LoQ (**Figure 2D**).

### 3.3 Rhizobiome bacterial profiling by 16S rRNA metataxonomics and metagenomics

**Figure 3:**
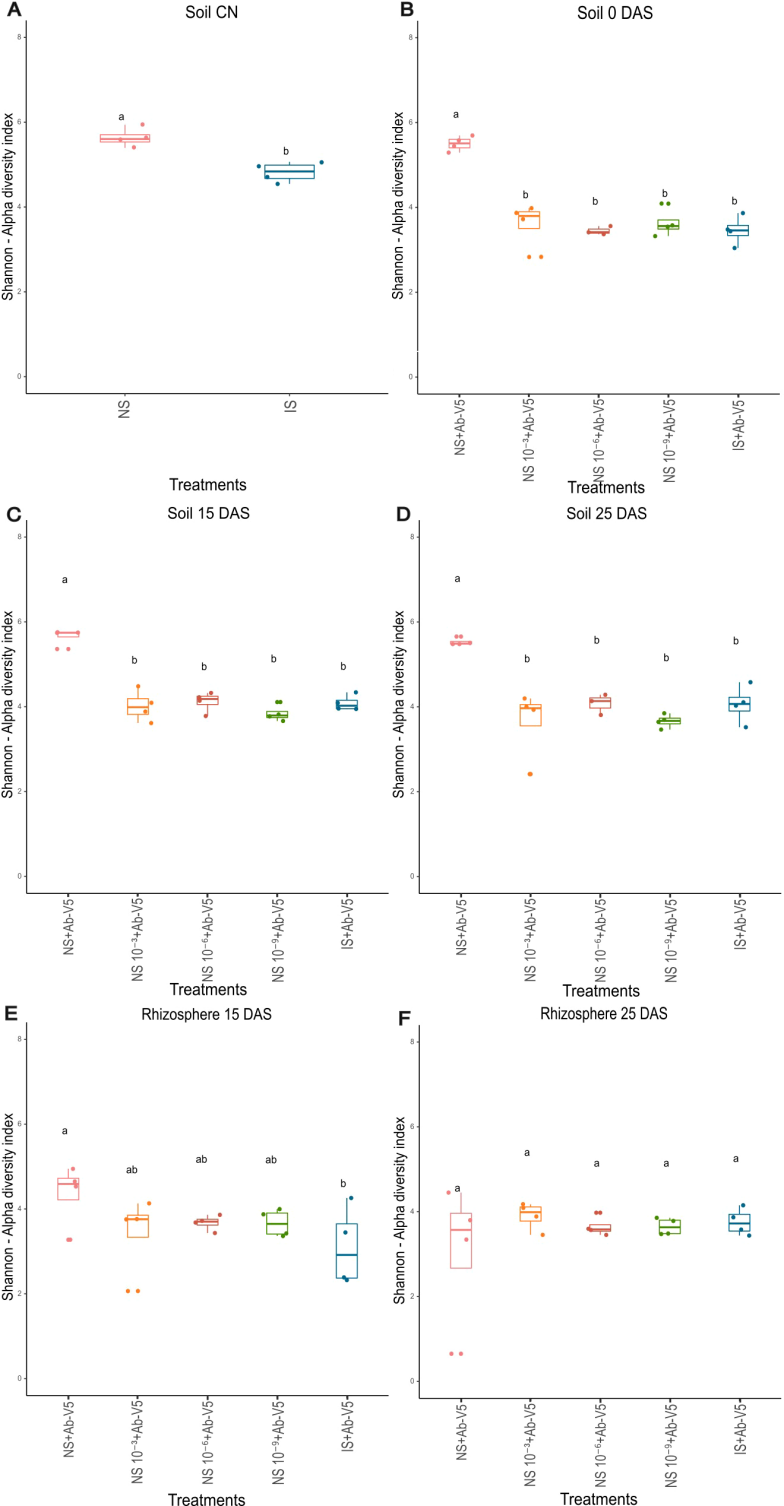
**(A–F)** Alpha diversity index Shannon from metataxonomic data of bulk soil and rhizosphere samples collected CN, 0, 15 and 25 DAS. Ab-V5, *Azospirillum brasilense* Ab-V5; NS + Ab-V5, Natural soil (microbial community); NS 10^−3^ + Ab-V5, dilution 10^−3^ of NS; NS 10^−6^ + Ab-V5, dilution 10^−6^ of NS; NS 10^−9^ + Ab-V5, dilution 10^−9^ of NS; IS, Irradiated soil.

Considering the bulk soil, Shannon’s alpha diversity was higher in the NS than in the IS treatment (**Figure 3A**). Alpha diversity in the three dilutions of soils NS + Ab-V5 as well as in the IS + Ab-V5 were not statistically different, and inferior to the NS + Ab-V5 at 0 DAS, 15 DAS and 25 DAS (**Figures 3B–D**). In rhizospheric samples collected at 15 DAS, the alpha diversity on treatment NS + Ab-V5 was significantly higher than IS + Ab-V5, all three dilutions were not statistically different from either the NS + Ab-V5, or the IS + Ab-V5 (LSD test, p-value < 0.05) (**Figure 3E**), and no significant differences among all treatments were observed at 25 DAS (**Figure 3F**).

Analysis of metataxonomics beta diversity revealed that bacterial communities in bulk and rhizospheric soils NS + Ab-V5 in all sampling times were separated from the other treatments (p-value < 0.05), while samples from the three dilution treatments of NS + Ab-V5, and IS + Ab-V5 were clustered in dimensional scale (**Supplementary Figure 1**).

**Figure 4:**
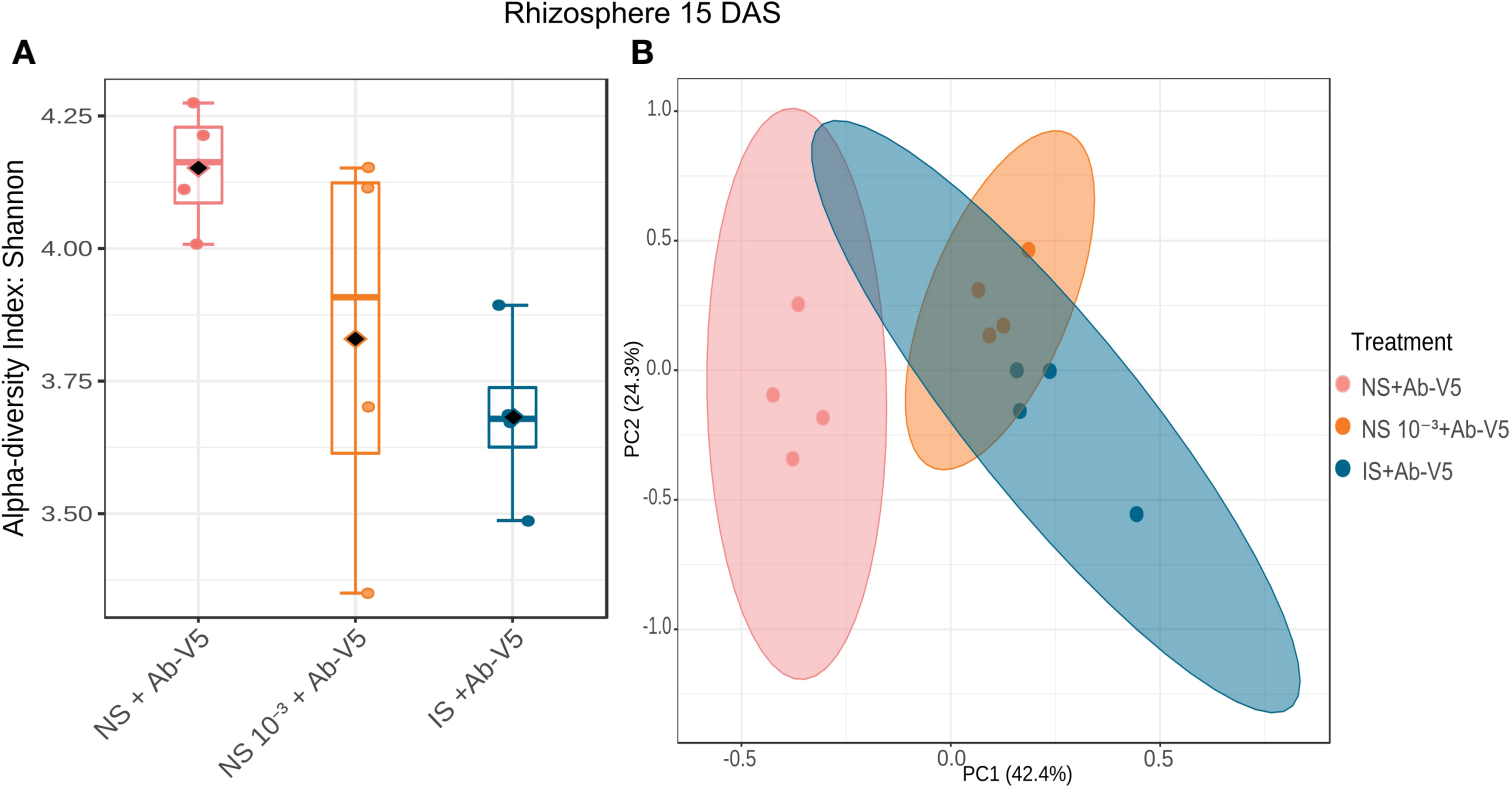
Alpha **(A)** and beta **(B)** diversity by metagenomics approach of rhizosphere samples collected 15 DAS. Ab-V5, *Azospirillum brasilense* Ab-V5; NS + Ab-V5, Natural soil (microbial community); NS 10^−3^ + Ab-V5, dilution 10^−3^ of NS; IS, Irradiated soil.

Similarly to metataxonomics analysis, metagenomics revealed significantly higher Shannon’s alpha diversity in NS + Ab-V5 rhizosphere samples compared to IS + Ab-V5, and both did not differ from the treatment NS 10^−3^ + Ab-V5 (LSD test, p-value < 0.05) (**Figure 4A**). Beta diversity bacterial communities in rhizosphere NS and IS + Ab-V5 were clustered separately by PCA, with only small intersection between their 95% confidence ellipses. The variance explained by PC1 distinguished treatments NS and NS 10 ^-3^ + Ab-V5 (p-value < 0.05). Samples from treatments NS 10^-3^ + Ab-V5 and IS + An-V5 were closely clustered in bidimensional scale (**Figure 4B**).

### 3.4 Network analysis

In order to investigate the ecological impact of Ab-V5 in the rhizosphere microbiome composition, we analyzed correlation networks of bulk soil and rhizosphere communities. Among bulk soil samples, the community in IS + Ab-V5 had the lowest and NS 10^−6^ + Ab-V5 had the highest number of nodes compared to the other treatments (**Supplementary Table 4**). NS 10^−6^ + Ab-V5 also showed the highest number of edges (i. e., connections among phylotypes), average number of neighbors, network diameter, clustering coefficient and network heterogeneity (**Supplementary Table 4**). Within rhizosphere samples, NS + Ab-V5 had the highest number of nodes, edges, neighbors, clustering coefficient, network density, heterogeneity and centralization (**Supplementary Table 4**).

### 3.5 Metagenomic analysis of nitrogen and phosphorus metabolism

We focused mainly on nitrogen and phosphorus metabolism to understand the ecological relevance of inoculation with *A. brasilense* Ab-V5 in three contrasting treatments: NS + Ab-V5, NS 10^−3^ + Ab-V5 and IS + Ab-V5, 15 DAS. These treatments were selected for metagenomics analysis based on plant phenotypic response to the dilution-to-extinction gradient created.

**Figure 5:**
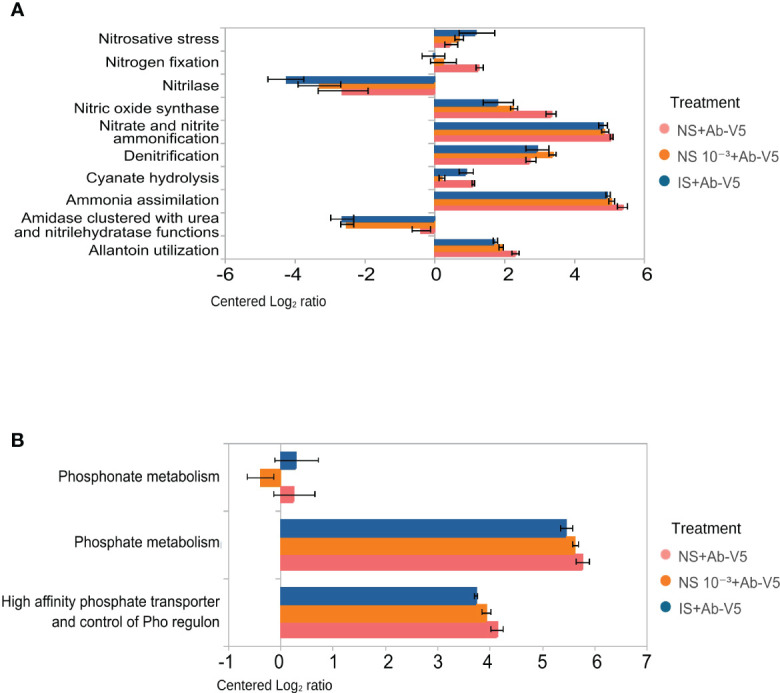
Metagenomic analysis of bacterial metabolism of nitrogen and phosphorus on NS + Ab-V5, NS 10^−3^ + Ab-V5 and IS + Ab-V5 rhizospheric soils, sampled after 15 days of maize seedling inoculated with PGPB *Azospirillum brasilense* Ab-V5. **(A)** Centered Log_2_ ratios for genes related to nitrogen metabolism. **(B)** Centered Log_2_ ratios for genes related to phosphorus metabolism. Only functions that change significantly among the soils are shown (p < 0.05; Kruskal-Wallis). NS + Ab-V5: Natural soil (microbial community); NS 10^−3^ + Ab-V5: dilution 10^−3^ of NS; NS 10^−6^ + Ab-V5: dilution 10^−6^ of NS; NS 10^−9^ + Ab-V5: dilution 10^−9^ of NS; IS: Irradiated soil.

Denitrification genes were significantly more abundant in NS 10^−3^ + Ab-V5 rhizospheric samples compared to other treatments, whereas NS + Ab-V5 rhizosphere samples had more genes related to nitrogen fixation, nitric oxide synthase, nitrate and nitrite ammonification, ammonia assimilation, allantoin utilization. The rhizosphere of IS + Ab-V5 was enriched with nitrosative stress, nitrilase and amidase clustered with urea and nitrile hydratase functions (**Figure 5A**).

Phosphate metabolism and high affinity phosphate transporter and control of PHO regulon were more abundant in NS + Ab-V5 and IS + Ab-V5 (**Figure 5B**). Other features from the metagenome data set were analyzed in order to search for important ecological information correlated with better persistence of Ab-V5 and plant growth promotion in rhizospheric soil constituted in the treatment of soil NS 10^−3^ + Ab-V5. Genes related to tryptophan catabolism, mannitol utilization, DNA replication, biosynthesis of UDP-N-acetylmuramate from frutose-6-phosphate were more abundant in the rhizosphere when plants were cultivated in soil amended with NS 10^−3^ + Ab-V5 (**Table 2**). Siderophore assembly, chitin and N-acetylglucosamine utilization, plasmid replication, DNA and RNA processing and transcription factors were enriched in both NS 10^−3^ + Ab-V5 and IS + Ab-V5 in comparison with NS + Ab-V5 (**Table 2**). On the other hand, sigma B stress response regulation was significantly more abundant in NS + Ab-V5 (**Table 2**).

**Table 2:** Comparison of metagenomic selected functions’ relative abundance in maize rhizosphere of treatments NS + Ab-V5, NS 10^-3^ + Ab-V5 and IS + Ab-V5 collected 15 days after seeding.

Functions	Centered Log_2_ Ratio (CLR) mean ± standard deviation		
	NS + Ab-V5	NS 10^−3^ + Ab-V5	IS + Ab-V5
Mannitol utilization	1.57 ± 0.07 b	2.06 ± 0.17 a	1.90 ± 0.39 ab
One carbon metabolism by tetrahydropterines	1.70 ± 0.12 a	1.73 ± 0.05 a	1.47 ± 0.16 b
Tryptophan catabolism	1.50 ± 0.05 b	1.69 ± 0.05 a	1.56 ± 0.11 b
UDP N-acetylmuramate from fructose-6-phosphate	2.57 ± 0.10 b	2.74 ± 0.06 a	2.56 ± 0.10 b
DNA replication cluster 1	2.54 ± 0.09 b	2.70 ± 0.08 a	2.53 ± 0.09 b
RNA processing and degradation	3.35 ± 0.07 b	3.51 ± 0.05 a	3.54 ± 0.09 a
Plasmid replication	1.19 ± 0.05 b	1.48 ± 0.15 a	1.38 ± 0.15 ab
Chitin and N-acetylglucosamine	3.22 ± 0.04 b	3.60 ± 0.14 a	3.47 ± 0.23 ab
PhoR-PhoB two-component regulatory system	1.55 ± 0.11 a	1.37 ± 0.06 b	1.24 ± 0.12 b
Siderophore assembly	1.40 ± 0.15 b	1.86 ± 0.13 a	1.89 ± 0.36 a
Transcription factors	3.94 ± 0.11 a	4.08 ± 0.09 a	3.95 ± 0.14 a
Sigma B stress response regulation	2.78 ± 0.16 a	2.14 ± 0.09 b	2.33 ± 0.32 b

Different letters indicate statistically significant mean differences among treatments according to LSD test (p-value < 0.05). Ab-V5, Azospirillum brasilense Ab-V5; NS + Ab V5, Natural soil (microbial community); NS 10^⁻³^ + Ab-V5, dilution 10^⁻³^ of NS; NS 10^⁻⁶^ + AbV5: dilution 10^⁻⁶^ of NS; NS 10^⁻⁹^ + Ab-V5, dilution 10^⁻⁹^ of NS; IS, Irradiated soil.

### 3.6 Data integration (16S rRNA metataxonomics and metagenomics)

**Figure 6:**
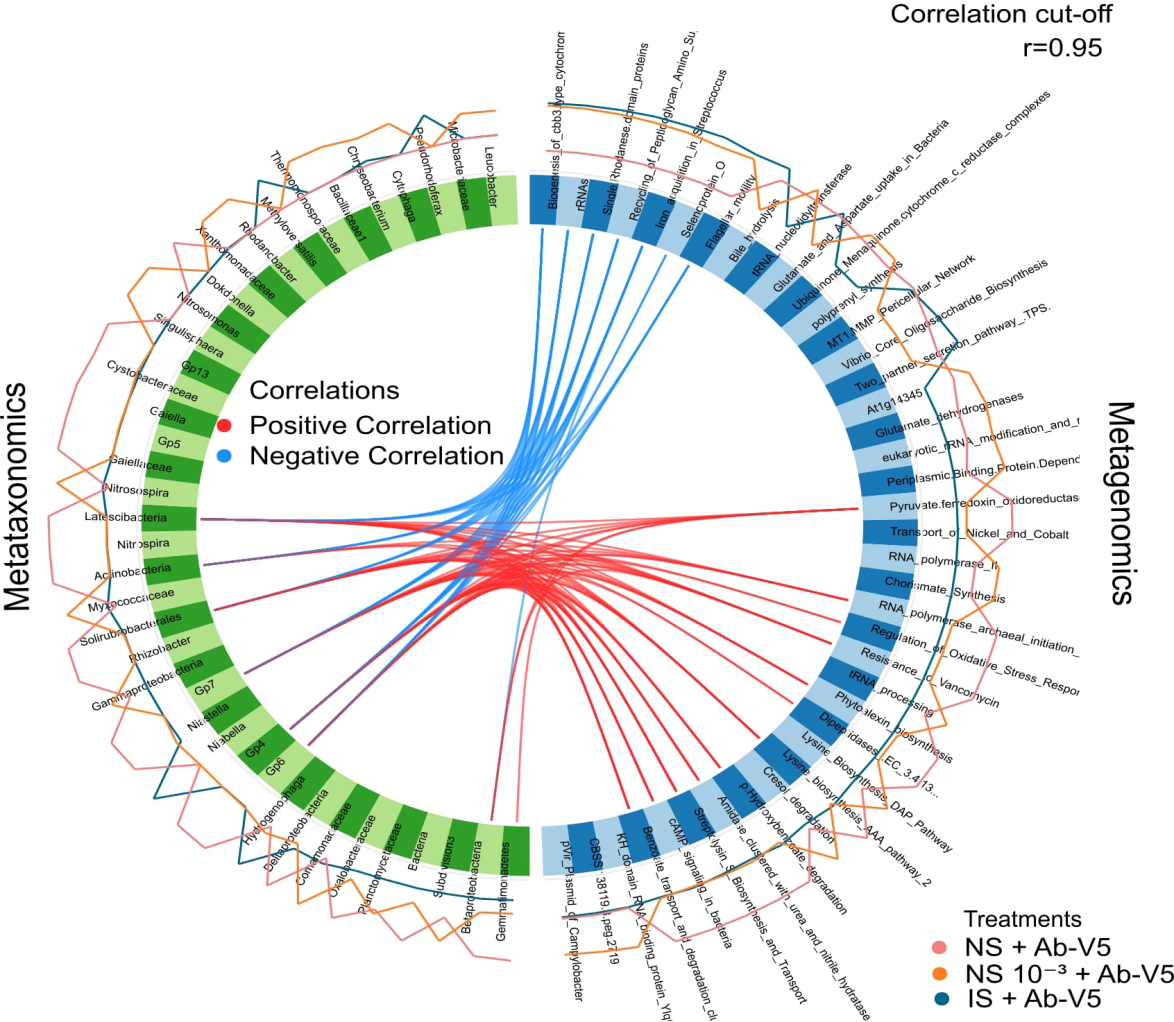
Correlation between different omics data sets from maize rhizospheric samples NS + Ab-V5, NS 10^-3^ + Ab-V5 and IS + Ab-V5. Ab-V5, Azospirillum brasilense Ab-V5; NS + Ab-V5, Natural soil (microbial community); NS 10^-3^ + Ab-V5, dilution 10^-3^ of NS; NS 10^-6^ + AbV5: dilution 10^-6^ of NS; NS 10^-9^ + Ab-V5, dilution 10^-9^ of NS; IS, Irradiated soil.

By integrating 16S rRNA metataxonomics and metagenomics data sets, several signatures were found to discriminate among rhizospheric microbial communities in the treatments NS + Ab-V5, NS 10^−3^ + Ab-V5 and IS + Ab-V5. Among these signatures from the two datasets, the correlation index was estimated at 98% (**Supplementary Figure 2**). A total of 19 taxa (not all assigned up to the genus level) were considered potential biomarkers that showed higher abundance in the NS + Ab-V5 rhizosphere samples (**Figure 6**). These taxa were identified as *Dokdonella* (Pseudomonadota; Gammaproteobacteria; Xanthomonadales; Rhodanobacteraceae), Acidobacteriota (Gp4, Gp5, Gp6, Gp7 and Gp13), Cystobacteraceae (Pseudomonadota; Deltaproteobacteria; Myxococcales; Cystobacterineae), *Gaiella* (Acidobacteriota; Rubrobacteria; Gaiellales; Gaiellaceae), other unclassified Gaiellaceae and Actinomycetota, Solirubrobacterales (Actinomycetota; Thermoleophilia), *Nitrospira* (Nitrospirota; Nitrospiria; Nitrospirales; Nitrospiraceae), *Rhizobacter* (Pseudomonadota; Betaproteobacteria; Burkholderiales; Burkholderiales), *Niastella* (Bacteroidota; Chitinophagia; Chitinophagales; Chitinophagaceae), Oxalobacteraceae (Pseudomonadota; Betaproteobacteria; Burkholderiales) and other Betaproteobacteria, Deltaproteobacteria, Latescibacteria (FCB group) and Gemmatimonadetes (FCB group; Gemmatimonadota). From this group of potential biomarkers of NS + Ab-V5, six taxa showed negative correlation with seven metagenomic features, all highly abundant in the IS + Ab-V5 and NS 10^−3^ + Ab-V5 rhizosphere samples: biogenesis of cbb3-type cytochrome, rRNAs, single rhodanese domain proteins, recycling of peptidoglycan, iron acquisition and selenoprotein. Whereas these taxa showed positive correlations with metagenomic features more abundant in the NS + Ab-V5 samples, some of which are related to environmental xenobiotics degradation, phytoalexin biosynthesis, streptolysin S biosynthesis and transport, cAMP signaling in bacteria and regulation of oxidative stress response (**Figure 6**).

Thirteen bacterial taxa were more abundant in the NS 10^−3^ + Ab-V5 rhizosphere samples: *Leucobacter* (Actinomycetota; Actinomycetes; Micrococcales; Microbacteriaceae), *Cytophaga* (Bacteroidota; Cytophagia; Cytophagales; Cytophagaceae), *Chryseobacterium* (Bacteroidota; Flavobacteriia; Flavobacteriales; Weeksellaceae), Bacillaceae (Bacillota; Bacilli; Bacillales), *Rhodanobacter* (Pseudomonadota; Gammaproteobacteria; Xanthomonadales; Rhodanobacteraceae), Xanthomonadaceae (Pseudomonadota; Gammaproteobacteria; Xanthomonadales), *Nitrosomonas* (Pseudomonadota; Betaproteobacteria; Nitrosomonadales; Nitrosomonadaceae), *Nitrosospira* (Betaproteobacteria; Nitrosomonadales; Nitrosomonadaceae), *Singulisphaera* (Planctomycetota; Planctomycetia; Isosphaerales; Isosphaeraceae), Myxococcaceae (Myxococcota; Myxococcia; Myxococcales; Cystobacterineae), Gammaproteobacteria, Comamonadaceae and Verrucomicrobia subdivision 3 (Verrucomicrobiota; Verrucomicrobiae; Verrucomicrobiales) (**Figure 6**). The taxa *Pseudorhodoferax* (Pseudomonadota; Betaproteobacteria; Burkholderiales; Comamonadaceae)*, Methyloversatilis* (Pseudomonadota; Betaproteobacteria; Nitrosomonadales; Sterolibacteriaceae)*, Hydrogenophaga* (Pseudomonadota; Betaproteobacteria; Burkholderiales; Comamonadaceae) and *Niabella* (Bacteroidota; Chitinophagia; Chitinophagales; Chitinophagaceae) were found to be potential biomarkers for IS + Ab-V5 rhizosphere samples. The metagenome features more abundant in NS 10^−3^ + Ab-V5 were mostly related to riboses and amino acids metabolism, and cresol degradation. Other 5 features were more abundant in IS + Ab-V5, including glutamate and aspartate uptake in bacteria, oligosaccharide biosynthesis and bacterial secretion pathway (**Figure 6**).

## 4 Discussion

Understanding PGPB-plant-microbiome interactions is vital to improve strain selection, inoculant formulation and technologies of application of bioproducts, and optimize the soil microbiome through agricultural practices (Volpiano et al., 2022). Most of the current knowledge about the rhizobiome is limited to microbial community composition, but little is known regarding PGPB-native soil microbial community function in association with host plants (Castellano-Hinojosa and Strauss, 2021; Wang et al., 2021). The performance and persistence of PGPB when applied in native microbial communities associated with seeds and soil is dependent on their interaction with the native microbial community and has caused concerns about the efficiency of promoting plant growth in field conditions, highly influenced by edaphoclimatic conditions (Orozco-Mosqueda et al., 2021). Advancements in omics platforms and integration with bioinformatics and statistical tools allow exploration of ecological and metabolic services the microbial community provides to favor the host plant. This can lead, among other benefits, to the selection of efficient bacterial strains and functions in promoting plant growth (Ramakrishna et al., 2019; Irineu et al., 2022; Monteoliva et al., 2022). This study expanded the current knowledge on these topics by integrating molecular data supported by plant phenomics in order to understand the performance and ecological impact of a native bacterial community on a commercial bioinoculant, *A. brasilense* Ab-V5, in the rhizosphere of a commercial maize hybrid.

Previous studies have shown that *A. brasilense* Ab-V5 has consistent results on field trials performed with maize (Barbosa et al., 2022; Hungria et al., 2022), and acts at the rhizosphere level. In field conditions with maize, Ab-V5 significantly increased plant height, stalk diameter, number of leaves and weight of 100 grains compared to non-inoculated control. When associated with the PGPB Bacillus thuringiensis RZ2MS9, Ab-V5 also positively affects the enrichment of the class Actinobacteria and the order Actinomycetales in maize rhizosphere (Ferrarezi et al., 2022). However, the strain Ab-V5 was not found in the microbiome of inoculated plants (Bender et al., 2022). Therefore, to investigate the soil in comparison with the rhizosphere microbiomes was important to advance in current knowledge, as the interactions occurring at the rhizosphere must control the response to inoculation. The dilution-to-extinction has proven to be successful in this approach confirming a range of effects on plant growth promotion affected by Ab-V5 inoculation. The natural soil (NS) represented the original state of the soil microbial community collected in a maize field, i. e. an usual condition encountered by an inoculant applied in crops and, by diluting it, we assembled new communities of beneficial microbes at the rhizosphere as a result of assortment and coadaptation overtime by the co-influence of plant-*A. brasilense*.

We observed that plant growth promotion parameters and qPCR-quantified abundance of Ab-V5 in the bulk soil and rhizosphere were lower in NS, compared to the other treatments. The NS + Ab-V5 community also showed the highest number of nodes and edges in the correlation network analysis, indicating high complexity interactions among members of the rhizobiome. Alpha and beta diversity analysis estimated both by metataxonomics and metagenomics profiling indicated that NS + Ab-V5 was separated from the other treatments, demonstrating the power of selected groups from NS in shaping new stable microbial communities that potentially helped Ab-V5 in promoting plant growth. Shade (2017) discussed the misconception of higher alpha diversity linked to success or failure of a microbial community, highlighting the importance of understanding the ecological mechanisms in a comparative context.

We observed that the higher diversity NS did not always reflect a better community for the plant-PGPB interaction. Chen et al. (2020) concluded that soil microbial diversity is important for the accumulation of plant biomass and provided experimental evidence of the main impact in microbiome functionality as a result of the removal of non-redundant species from the ecosystem,. It is known that the removal of some species, for instance after diluting a natural microbial community, leads to lower interspecific competition, triggering microbial recolonization and more intensive processes of reassembly of whole communities (Chen et al., 2020). From our results, the lower alpha-diversity observed in IS and dilutions of NS compared to the original NS in bulk soil (0, 15 and 25 DAS) and rhizospheric samples (15 DAS) with a stabilization in rhizospheric samples 25 DAS suggests that the removal of species from NS by diluting its microbial solution decreased interspecific microbial competition and triggered deterministic and stochastic processes of recolonization of plant-PGPB-associated niches. Compensated by the functional redundancy of species as a result of deterministic and stochastic processes during reassembly of soil microbiomes influenced by plant-PGPB.The overall better performance of NS dilutions 10^-3^, 10^−6^, 10^−9^ and IS + Ab-V5 is probably the result of a competitive process in which specific soil native beneficial microbial groups and functions were recruited along the process of microbial recolonization, contributing to enhance Ab-V5 inoculation performance and plant growth parameters compared to those grown in undiluted NS. Thus, understanding the complex PGPB-holobiont and diversity is vital to manage agriculture in a more sustainable way through the use of strategies that maximizes the inoculant potential (Trabelsi and Mhamdi, 2013). Future studies should be carried out to clarify the best agricultural practices needed to modify the native soil microbiome and promote microbial groups/functions that might help optimize the results after application of inoculants such as Ab-V5. The community coalescence methodology described by Huet et al. (2023) is a feasible way to better understand how to restore impaired functions in soils under common agricultural treatments, such as pesticides and mineral fertilizers, both before and after application of bioproducts.

Interestingly, the abundance of phosphorus metabolism-related genes was significantly higher in the NS + Ab-V5 rhizosphere metagenome. This could be explained by the initial difference in soil P content, 96 mg.dm^−3^ at the NS treatment, and 722 mg.dm^−3^ in the IS, impacting microbial selection for P solubilization in the NS. Genes related to BNF (biological nitrogen fixation) – denitrification- were also higher in the NS + Ab-V5 treatment, and could have influenced plant growth performance. These potential functions can be tracked by gene markers in the environment by metagenomics, indicating how nitrogen fixation, nitrite and nitrate ammonification or ammonia assimilation change in different situations. As well, to identify shifts of gene markers to phosphate metabolism or the Pho regulon, a regulatory mechanism for cellular management of inorganic phosphate (Santos-Beneit, 2015).

The undiluted NS was significantly less responsive to inoculation with Ab-V5 compared to dilutions 10^−3^, 10^−6^, 10^−9^ and IS with no addition of NS microbiome, and it reflected in plant growth-promotion parameters. The higher abundance of Ab-V5 quantified by qPCR in dilutions 10^−3^ and 10^−6^ of NS and IS suggested an effect of niche occupation by the inoculant. More specifically in NS 10^−3^ and NS 10^−6^, we hypothesize that there might be a joint effect between specific elements in the soil native community and the PGPB, impacting the inoculant dynamics. By diluting the soil native microbial community, it is expected that the proportion of the most competitive strains decreases, making more ecological niches available for invasive strains such as Ab-V5 to colonize the host. Similar results were reported by Alberton et al. (2006), when evaluating the effects of soil dilution in rhizobia nodulation of common beans and soybean.

From metagenomics and metataxonomics data integration, we identified genes responsible for (hydroxy)benzoate degradation, phytoalexin biosynthesis, cAMP signaling and regulation of oxidative stress response. These genes were positively correlated to six taxa more abundant in NS + Ab-V5, including three groups of *Acidobacteria*, *Solirubrobacterales*, *Actinobacteria* and *Latescibacteria*. This indicates that some microbiome members have important functions for detoxification from xenobiotic compounds and stress tolerance in this treatment, though impacting plant growth promotion status and PGPB persistence in the microbiome as supported by plant phenotyping and qPCR data. *Acidobacteria* was recently described in a BTEX (benzene, toluene, ethylbenzene and xylene) biodegraders-based consortium enriched from a petroleum-contaminated soil (Eze, 2021), while Solirubrobacterales was identified in low abundance in petroleum refinery sludge communities (Sarkar et al., 2016). We also identified bacteria taxa more abundant in NS + Ab-V5 negatively correlated with genes related to flagellar motility, selenoprotein, iron acquisition, recycling of peptidoglycan and biogenesis of cbb3-type cytochrome c oxidase (cbb_3_-Cox), which were more abundant in IS + Ab-V5 and NS 10^−3^ + Ab-V5. These pathways have been described as important regulators of nitrogen and CO_2_ fixation (cbb_3_-Cox; Ekici et al., 2012), selenium uptake (selenoprotein; Ye et al., 2020), bacterial chemotaxis (flagellar motility; Colin et al., 2021) and survivability (recycling of peptidoglycan; Borisova et al., 2016). The capacity of using a variety of compounds present in root exudates has been linked to the colonization and prevalence strategy of two different *Azospirillum* sp. Strains (Gao et al., 2022). From the plant´s point-of view, the effects of *Herbaspirillum seropedicae* on N uptake and assimilation machinery, as well as carbon/nitogen metabolism in maize was also investigated through plant transcriptomic-proteomic integration approach (Irineu et al., 2022).

Altogether, these pieces of evidence strongly suggest that the role of *A. brasilense* Ab-V5 as PGPB is dependent on interactions with soil native microbial groups. Beneficial bacteria taxa found in the rhizosphere, such as *Devosia*, *Stenotrophomonas* and *Pseudoxanthomonas*, have been reported as potential for a range of agri-biotechnological applications. *Devosia* spp. has been reported as a potential plant growth promoter by fixing nitrogen and alleviating abiotic stresses (Peng et al., 2021). *Stenotrophomonas* spp. have an important ecological role in the N and S cycles and several strains of this genus can engage in beneficial interactions in plant growth promotion and protection (Ryan et al., 2009). Strains of *Pseudoxanthomonas* spp. and *Stenotrophomonas* spp. with nematostatic and nematocidal activities, and arsenic resistance effects as plant growth promoters has been reported from plant-associated soils (Huang et al., 2009; Hu et al., 2019; Huda et al., 2022). Soil microbiological health attributes such as utilization of different sources of carbon, nucleic acids processing and nutrient cycling were targeted as good metagenomic indicators for PGPB performance in field conditions.

Currently, the use of multi-strain consortia to assemble synthetic communities (SynCom) with the purpose of enhancing plant growth has been proposed. However, the methods used to select and predict the output of these strains in field conditions are poorly understood. Although it was not tested in this study, we propose the use of multi-omics data integration as a complementary tool to select microbiome functions and taxa that have potential to be incorporated in SynCom. Future studies should be carried out to better understand the impact of optimizing the soil microbiome and, consequently, manipulating the holobiome to improve the effects of microbial products through sustainable crop management practices, such as crop rotation, organic fertilization, use of biological agents, and no-tillage (Monteoliva et al., 2022).

## Code availability

Sequencing data quality control and processing for metataxonomics: HackMD (https://hackmd.io/@W60ke7XaTbyWRewvltUAtg/rkFHmk9hF).

## Data availability statement

The data presented in the study are deposited in the European Nucleotide Archive (ENA) repository (https://www.ebi.ac.uk/ena), accession numbers PRJEB60483 and PRJEB61641.

## Author contributions

JF: Formal analysis, Investigation, Methodology, Data curation, Writing – original draft, review and editing. HD: Investigation, Methodology, Writing - review and editing; LS: Data curation, Writing – review and editing. JA: Conceptualization, Funding Acquisition. MH: Writing - review and editing MQ: Conceptualization, Supervision, Funding acquisition, Writing – review and editing. All authors contributed to the article and approved the submitted version.
