# Supplementary material for: Meta-omics integration approach reveals the effect of soil native microbiome diversity in the performance of inoculant Azospirillum brasilense
Source: Front Plant Sci. 2023 Jun 29;14:1172839. doi: 10.3389/fpls.2023.1172839 (PMC10340089; doi:10.3389/fpls.2023.1172839)

***Supplementary Material***

**Supplementary Table 1.** Fertility analysis of samples of Irradiated Soil (IS) and Natural Soil (NS) used to construct native microbial community gradient.

| **Type of analysis** | **Attribute** | **Unit** | **IS** | **NS** |
| --- | --- | --- | --- | --- |
| **Physical** | Sand | g/kg | 554 | 416 |
|  | Silt | g/kg | 94 | 251 |
|  | Clay | g/kg | 353 | 333 |
|  | Texture class | - | Clay | clay loam |
| **Chemical** | B hot water | mg/dm³ | 0.97 | 0.3 |
|  | Cu DTPA | mg/dm³ | 4.4 | 2.6 |
|  | Fe DTPA | mg/dm³ | 59 | 38 |
|  | Mn DTPA | mg/dm³ | 27.8 | 42.6 |
|  | Zn DTPA | mg/dm³ | 20.4 | 5.9 |
|  | pH CaCl_2_ | - | 6.3 | 5.7 |
|  | M,O, colorimetric | g/dm³ | 55 | 16 |
|  | P resin | mg/dm³ | 722 | 96 |
|  | S (Ca_3_(PO_4_)_2_) | mg/dm³ | 51 | <10 |
|  | K resin | mmolc/dm³ | 12.1 | 4.6 |
|  | Ca resin | mmolc/dm³ | 223 | 41 |
|  | Mg resin | mmolc/dm³ | 51 | 20 |
|  | Al KCl 1 mol/L | mmolc/dm³ | 2 | 1 |
|  | H+Al SMP | mmolc/dm³ | 16 | 25 |
|  | Base Saturation | mmolc/dm³ | 286.1 | 65.6 |
|  | Capacity of Cations Exchange | mmolc/dm³ | 302.1 | 90.6. |
|  | V | % | 95 | 72 |
|  | m | % | 1 | 2 |

**Supplementary Table 2.** Description of samples for metataxonomics 16S rRNA and metagenomics.

| **Sample type** | **Collection time** | **Treatment** | **Description** | **Omics** | **Number of samples** | **Sample identification** |
| --- | --- | --- | --- | --- | --- | --- |
| Bulk soil | CN | NS + Ab-V5 | Native soil (NS) microbial community used to construct a microbial community diversity gradient | Metataxonomics 16S rRNA | 4 | SA-CN |
| Bulk soil | CN | IS +Ab-V5 | Soil sterilized by gamma-irradiation | Metataxonomics 16S rRNA | 4 | SE-CN |
| Bulk soil | 0 DAS | NS + Ab-V5 | Native soil (NS) microbial community used to construct a microbial community diversity gradient amended with Ab-V5 | Metataxonomics 16S rRNA | 4 | SA-0DAS |
| Bulk soil | 0 DAS | NS 10^-3^ + Ab-V5 | Dilution 10^-3^ of NS amended with Ab-V5 | Metataxonomics 16S rRNA | 4 | SB-0DAS |
| Bulk soil | 0 DAS | NS 10^-6^ + Ab-V5 | Dilution 10^-6^ of NS amended with Ab-V5 | Metataxonomics 16S rRNA | 4 | SC-0DAS |
| Bulk soil | 0 DAS | NS 10^-9^ +Ab-V5 | Dilution 10^-9^ of NS amended with Ab-V5 | Metataxonomics 16S rRNA | 4 | SD-0DAS |
| Bulk soil | 0 DAS | IS +Ab-V5 | Soil sterilized by gamma-irradiation amended with Ab-V5 | Metataxonomics 16S rRNA | 4 | SE-0DAS |
| Bulk soil | 15 DAS | NS + Ab-V5 | Native soil (NS) microbial community used to construct a microbial community diversity gradient amended with Ab-V5 | Metataxonomics 16S rRNA | 4 | SA-15DAS |
| Bulk soil | 15 DAS | NS 10^-3^ + Ab-V5 | Dilution 10^-3^ of NS amended with Ab-V5 | Metataxonomics 16S rRNA | 4 | SB-15DAS |
| Bulk soil | 15 DAS | NS 10^-6^ + Ab-V5 | Dilution 10^-6^ of NS amended with Ab-V5 | Metataxonomics 16S rRNA | 4 | SC-15DAS |
| Bulk soil | 15 DAS | NS 10^-9^ +Ab-V5 | Dilution 10^-9^ of NS amended with Ab-V5 | Metataxonomics 16S rRNA | 4 | SD-15DAS |
| Bulk soil | 15 DAS | IS +Ab-V5 | Soil sterilized by gamma-irradiation amended with Ab-V5 | Metataxonomics 16S rRNA | 4 | SE-15DAS |
| Bulk soil | 25 DAS | NS + Ab-V5 | Native soil (NS) microbial community used to construct a microbial community diversity gradient amended with Ab-V5 | Metataxonomics 16S rRNA | 4 | SA-25DAS |
| Bulk soil | 25 DAS | NS 10^-3^ + Ab-V5 | Dilution 10^-3^ of NS amended with Ab-V5 | Metataxonomics 16S rRNA | 4 | SB-25DAS |
| Bulk soil | 25 DAS | NS 10^-6^ + Ab-V5 | Dilution 10^-6^ of NS amended with Ab-V5 | Metataxonomics 16S rRNA | 4 | SC-25DAS |
| Bulk soil | 25 DAS | NS 10^-9^ +Ab-V5 | Dilution 10^-9^ of NS amended with Ab-V5 | Metataxonomics 16S rRNA | 4 | SD-25DAS |
| Bulk soil | 25 DAS | IS +Ab-V5 | Soil sterilized by gamma-irradiation amended with Ab-V5 | Metataxonomics 16S rRNA | 4 | SE-25DAS |
| Rhizosphere | 15 DAS | NS + Ab-V5 | Native soil (NS) microbial community used to construct a microbial community diversity gradient amended with Ab-V5 | Metataxonomics 16S rRNA  Metagenomics | 4  4 | RiA-15DAS |
| Rhizosphere | 15 DAS | NS 10^-3^ + Ab-V5 | Dilution 10^-3^ of NS amended with Ab-V5 | Metataxonomics 16S rRNA  Metagenomics | 4  4 | RiB-15DAS |
| Rhizosphere | 15 DAS | NS 10^-6^ + Ab-V5 | Dilution 10^-6^ of NS amended with Ab-V5 | Metataxonomics 16S rRNA | 4 | RiC-15DAS |
| Rhizosphere | 15 DAS | NS 10^-9^ +Ab-V5 | Dilution 10^-9^ of NS amended with Ab-V5 | Metataxonomics 16S rRNA | 4 | RiD-15DAS |
| Rhizosphere | 15 DAS | IS +Ab-V5 | Soil sterilized by gamma-irradiation amended with Ab-V5 | Metataxonomics 16S rRNA  Metagenomics | 4  4 | RiE-15DAS |
| Rhizosphere | 25 DAS | NS + Ab-V5 | Native soil (NS) microbial community used to construct a microbial community diversity gradient amended with Ab-V5 | Metataxonomics 16S rRNA | 4 | RiA-25DAS |
| Rhizosphere | 25 DAS | NS 10^-3^ + Ab-V5 | Dilution 10^-3^ of NS amended with Ab-V5 | Metataxonomics 16S rRNA | 4 | RiB-25DAS |
| Rhizosphere | 25 DAS | NS 10^-6^ + Ab-V5 | Dilution 10^-6^ of NS amended with Ab-V5 | Metataxonomics 16S rRNA | 4 | RiC-25DAS |
| Rhizosphere | 25 DAS | NS 10^-9^ +Ab-V5 | Dilution 10^-9^ of NS amended with Ab-V5 | Metataxonomics 16S rRNA | 4 | RiD-25DAS |
| Rhizosphere | 25 DAS | IS +Ab-V5 | Soil sterilized by gamma-irradiation amended with Ab-V5 | Metataxonomics 16S rRNA | 4 | RiE-25DAS |

Note: CN - when natural soil (NS) microbial community gradient (dilutions 10⁻³, 10⁻⁶ and 10⁻⁹) was transferred to irradiated soil (IS); 15 and 25 DAS - days after sowing maize seeds inoculated with *Azospirillum brasilense* Ab-V5.

**Supplementary Table 3.** Summary of read counts of input, after quality filtering, denoising, merging and removing chloroplasts and mitochondria reads of metataxonomics 16S rRNA gene.

| **Sample** | **Input** | **Filtered** | **Denoised (Forward)** | **Denoised (Reverse)** | **Merged reads** | **Non-chimeric** | **Non-plastidial** |
| --- | --- | --- | --- | --- | --- | --- | --- |
| RiA-15DAS | 131808 | 113497 | 101335 | 97915 | 77521 | 56078 | 52240 |
| RiB-15DAS | 158162 | 143495 | 136130 | 135770 | 118571 | 79554 | 73358 |
| RiC-15DAS | 144744 | 130863 | 125083 | 124421 | 112601 | 68759 | 53317 |
| RiD-15DAS | 155115 | 141060 | 133840 | 133240 | 118606 | 76186 | 67908 |
| RiE-15DAS |  |  |  |  |  |  |  |
| RiA-25DAS | 140529 | 127567 | 117783 | 115422 | 95472 | 63427 | 61126 |
| RiB-25DAS | 129855 | 118081 | 111593 | 110788 | 96007 | 61869 | 53269 |
| RiC-25DAS | 139267 | 127510 | 121299 | 120474 | 108664 | 68018 | 60707 |
| RiD-25DAS | 141975 | 130411 | 124263 | 123305 | 111269 | 68946 | 61847 |
| RiE-25DAS |  |  |  |  |  |  |  |
| SA-CN | 84376 | 75319 | 64107 | 61065 | 37936 | 35103 |  |
| SE-CN | 91268 | 81277 | 72549 | 70454 | 53030 | 40765 |  |
| SA-0DAS | 92884 | 82940 | 72111 | 69153 | 44353 | 40291 |  |
| SB-0DAS | 82163 | 74962 | 69060 | 68132 | 57404 | 40837 |  |
| SC-0DAS | 86918 | 79649 | 75478 | 75037 | 67504 | 37409 |  |
| SD-0DAS | 88563 | 81368 | 76621 | 75628 | 67616 | 45438 |  |
| SE-0DAS | 86998 | 80578 | 75294 | 74677 | 65335 | 43876 |  |
| SA-15DAS | 98668 | 88482 | 76566 | 73823 | 45699 | 41775 |  |
| SB-15DAS | 84737 | 77424 | 70839 | 69091 | 56177 | 41297 |  |
| SC-15DAS | 91538 | 82764 | 76891 | 76143 | 65021 | 42534 |  |
| SD-15DAS | 88356 | 79800 | 73276 | 72440 | 61544 | 44273 |  |
| SE-15DAS | 98893 | 89537 | 83040 | 81408 | 68842 | 49384 |  |
| SA-25DAS | 83383 | 74309 | 65320 | 63355 | 43651 | 34113 |  |
| SB-25DAS | 82320 | 73738 | 68272 | 67340 | 55914 | 38697 |  |
| SC-25DAS | 88606 | 80014 | 74124 | 73471 | 45680 | 32231 |  |
| SD-25DAS | 89673 | 80924 | 74600 | 73450 | 61253 | 43704 |  |
| SE-25DAS | 86472 | 77306 | 71376 | 69822 | 57253 | 42443 |  |

**Supplementary Table 4.** Summary statistics of network correlation analysis of bulk and rhizospheric soil 16S rDNA metataxonomics by treatment.

|  | **Bulk soil** | | | | | **Rhizosphere** | | | | |
| --- | --- | --- | --- | --- | --- | --- | --- | --- | --- | --- |
| **Summary statistics** | **NS + Ab-V5** | **NS 10⁻³ + Ab-V5** | **NS 10⁻⁶ + Ab-V5** | **NS 10⁻⁹ + Ab-V5** | **IS + Ab-V5** | **NS + Ab-V5** | **NS 10⁻³ + Ab-V5** | **NS 10⁻⁶ + Ab-V5** | **NS 10⁻⁹ + Ab-V5** | **IS + Ab-V5** |
| **Number of nodes** | 122 | 125 | 165 | 138 | 116 | 178 | 147 | 106 | 139 | 165 |
| **Number of edges** | 642 | 872 | 1330 | 1044 | 852 | 4132 | 868 | 632 | 954 | 1232 |
| **Average number of neighbors** | 5.262 | 6.976 | 8.061 | 7.565 | 7.345 | 23.213 | 5.905 | 5.962 | 6.863 | 7.546 |
| **Network diameter** | 8 | 6 | 7 | 5 | 6 | 7 | 6 | 7 | 8 | 6 |
| **Network radius** | 5 | 4 | 4 | 4 | 4 | 5 | 3 | 4 | 5 | 4 |
| **Characteristic path length** | 3.608 | 3.034 | 2.985 | 2.900 | 2.955 | 2.86 | 3.044 | 3.387 | 3.333 | 3.216 |
| **Clustering coefficient** | 0.319 | 0.285 | 0.415 | 0.361 | 0.350 | 0.513 | 0.351 | 0.384 | 0.368 | 0.404 |
| **Network density** | 0.043 | 0.056 | 0.049 | 0.055 | 0.064 | 0.131 | 0.040 | 0.057 | 0.050 | 0.047 |
| **Network heterogeneity** | 0.526 | 0.500 | 1.047 | 0.644 | 0.468 | 0.953 | 0.898 | 0.516 | 0.665 | 0.899 |
| **Network centralization** | 0.057 | 0.090 | 0.333 | 0.129 | 0.103 | 0.359 | 0.334 | 0.088 | 0.141 | 0.209 |
| **Connected components** | 1 | 1 | 1 | 1 | 1 | 1 | 1 | 1 | 1 | 2 |

**Supplementary Table 5.** Summary of pre- and post-assembly and post-QC metagenome sequences - KBase metaSPAdes (Phred type: 33).

|  | **NS + Ab-V5 (r=4)** | **NS 10⁻³ + Ab-V5 (r=4)** | **IS +Ab-V5 (r=4)** |
| --- | --- | --- | --- |
| Total number of reads | 162,128,024 | 155,713,274 | 151,187,390 |
| Mean quality and standard deviation | 33.7 (2.04) | 33.7 (2.03) | 33.7 (2.06) |
| Mean read length and standard deviation | 98.3 (9.1) | 98.2 (9.4) | 98.2 (9.5) |
| Total number of reads after QC | 139,812,840 | 136,449,838 | 132,508,682 |
| GC content after QC | 64.1% | 61.7% | 61.2% |
| # contigs | 12,686 | 47,341 | 43,365 |
| # contigs (>= 1000 bp) | 12,686 | 47,341 | 43,365 |
| # contigs (>= 10000 bp) | 945 | 2,767 | 2,435 |
| # contigs (>= 100000 bp) | 22 | 28 | 137 |
| Largest contig | 261,132 | 388,525 | 941,264 |
| Total length | 60,705,612 | 217,158,537 | 218,468,206 |
| N50 | 5,502 | 4,958 | 5,500 |
| N75 | 2,919 | 3,010 | 3,057 |
| L50 | 2,296 | 10,599 | 7,086 |
| L75 | 6,267 | 24,949 | 20,904 |
| GC (%) assembly | 61.24 | 62.2 | 61.22 |
| Number of features (Prokka annotation) | 128,341 | 437,400 | 445,190 |
| Number of contigs (Prokka annotation) | 12,686 | 47,341 | 43,365 |

Note: NS: Natural soil (microbial community); NS 10⁻³: dilution 10⁻³ of NS; NS 10⁻⁶: dilution 10⁻⁶ of NS; NS 10⁻⁹: dilution 10⁻⁹ of NS; IS: Irradiated soil; Ab-V5: *Azospirillum brasilense* Ab-V5.


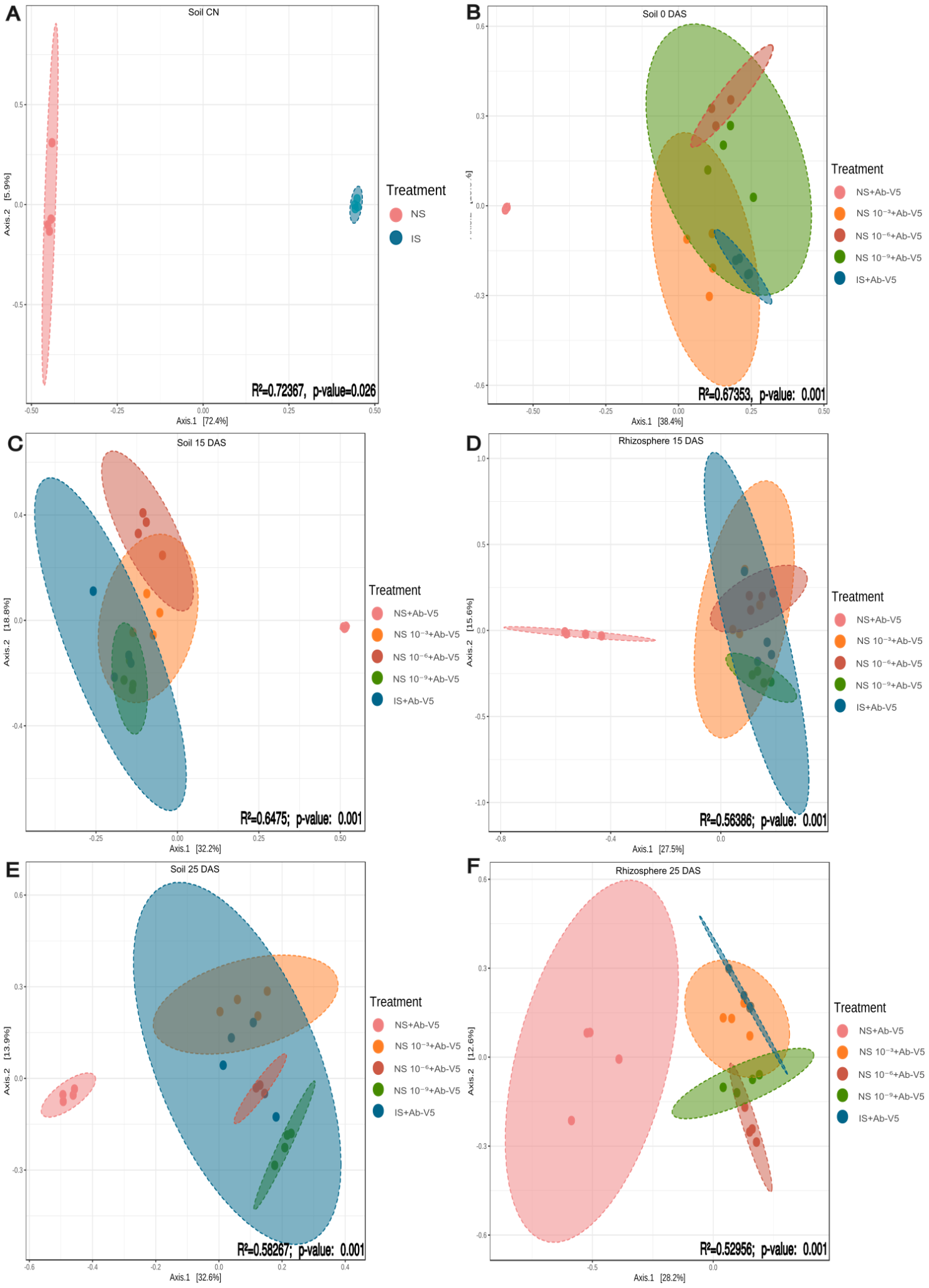


**Supplementary Figure 1.** Beta diversity 16S rRNA gene metataxonomic profiling of bulk soil (A-D) and rhizosphere samples (E-F) collected at CN, 0, 15 and 25 DAS. NS + Ab-V5: Natural soil (microbial community); NS 10⁻³ + Ab-V5: dilution 10⁻³ of NS; NS 10⁻⁶ + Ab-V5: dilution 10⁻⁶ of NS; NS 10⁻⁹ + Ab-V5: dilution 10⁻⁹ of NS; IS: Irradiated soil. Ab-V5: *Azospirillum brasilense* strain Ab-V5.

**Supplementary Figure 2.** Correlation between metagenomics and 16S rRNA metataxonomics datasets. NS + Ab-V5: Natural soil (microbial community); NS 10⁻³ + Ab-V5: dilution 10⁻³ of NS; IS: Irradiated soil. Ab-V5: *Azospirillum brasilense* strain Ab-V5.
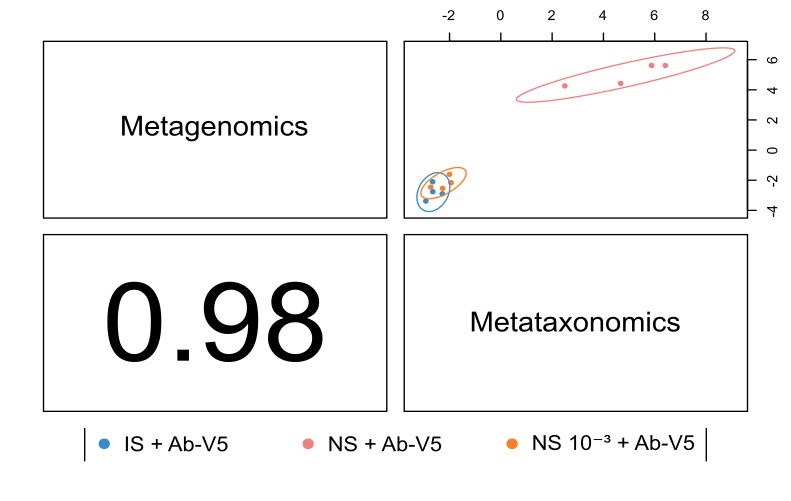

Supplement: Supplementary file 1 [file DataSheet_1.docx]
